# Food Consciousness Intervention Improves Interoceptive Sensitivity and Expression of Exteroception in Women

**DOI:** 10.3390/nu14030450

**Published:** 2022-01-20

**Authors:** Carina Carlucci Palazzo, Barbara Esteves Leghi, Rosa Wanda Diez-Garcia

**Affiliations:** 1 Internal Medicine Department, Ribeirão Preto Medicine School, University of São Paulo (USP), Ribeirão Preto 14049-900, Brazil; carinacp@usp.br (C.C.P.); barbara.leghi@usp.br (B.E.L.); 2 Laboratory of Food Practices and Behaviour (PratiCA), University of São Paulo (USP), Ribeirão Preto 14049-900, Brazil; 3 Department of Health Sciences, Ribeirão Preto Medicine School, University of São Paulo (USP), Ribeirão Preto 14049-900, Brazil

**Keywords:** food and nutrition education, nutritional trial, interoception, exteroception, text production, consciousness

## Abstract

The perception of the body’s internal state (interoception) and the perception and processing of environmental sensory stimuli (exteroception) act together to modulate adaptive behaviour, including eating behaviour, and are related to bodyweight control. This study evaluated the impact of the Food and Nutrition Education Program with Sensory and Cognitive Exercises on interoceptive sensitivity and on the expression of exteroceptive perception in women who experienced difficulty in controlling their body weight. Thirty-seven women were randomized into two groups and evaluated at two moments: before and after the intervention or before and after a 3- to 4-week waiting period. A heartbeat tracking task was used for interoception evaluation. Participants were asked to write a text describing three foods after tasting them for exteroception evaluation. After the intervention, the participants showed an increase in interoceptive sensitivity, and an increase in the expression of exteroceptive stimuli perception through a semantic assessment of their writing related to the tasting experience. In addition, the results point to a possible connection between the mechanisms governing interoception and exteroception. This work brings important contributions to the search for strategies capable of promoting the perception and integration of physiological and environmental stimuli in food consumption.

## 1. Introduction

Traditional strategies to control body weight, involving dietary restrictions and counting calories, have been considered to be ineffective in the medium and long terms [1]. Thus, there is currently a demand for the development of alternative strategies that can promote better eating behaviours, but which are not based on the practice of diets [2]. In this context, interventions that promote the perception of the sensory aspects of eating experiences have been identified as promising because they possibly point out ways to prevent and control obesity [3,4].

The way we perceive, interpret and react to the world around us happens through bodily sensations [5]. The sensations triggered by external stimuli (environment), and the internal pathways that these sensations impact, are known as exteroception [6], while the sensations triggered by internal stimuli, related to the body’s physiological state, are known as interoception. The brain constantly carries out the integration between exteroceptive and interoceptive stimuli, reflecting our adaptive ability to respond to environmental changes [7]. This adaptive response to environmental stimuli is favoured by the individual’s ability to consciously perceive subtle bodily changes resulting from such stimuli at the time that they occur [8].

The joint action of exteroceptive and interoceptive stimuli in the modulation of human behaviour is also important in eating behaviour [6]. In this case, interoceptive signals act to ensure body homeostasis, in a complex chain that involves metabolic and endocrine markers that act in the hypothalamic region of the brain and control hunger and satiety signs and thus contribute to energy balance [9]. Exteroceptive signals, on the other hand, act in the hedonic control of food consumption. Sensory stimuli (smell, vision, touch, taste, hearing and somatosensory signals) are processed together in the brain, triggering memories, expectations and also reward mechanisms that translate into food impulse/desire or refusal [3]. The homeostatic and hedonic controls of eating behaviour act synergistically in the construction of a complex representation of food in order to modulate the reward properties and affective value associated with specific foods at a given time [10,11,12], so that the pleasure experienced during food consumption is more intense in situations of food deprivation, which is known as “positive alliesthesia” [13], and less intense in monotonous diets, with little variation in available foods [14], or in contexts associated with negative values and feelings built during past eating experiences [15].

The balance between homeostatic and hedonic control in food consumption can be disrupted in some situations [16]. Changes in sensitivity to interoceptive signals with reduced perception of satiety signals, for example, can lead to increased positive alliesthesia and consequently to food overconsumption [4]. It is known that individuals with overweight or obesity tend to be less accurate in the perception of interoceptive signs; that is, they have lower interoceptive sensitivity [17,18] and have more accentuated reactions to the exteroceptive stimuli of food consumption, which can lead to exaggerated food consumption [19]. On the other hand, anorexia nervosa is also associated with lower interoceptive sensitivity, which in this case is manifested by the difficulty of perceiving signs of hunger [20]. Other eating disorder conditions, such as binge eating and bulimia, are associated with lower interoceptive awareness, that is, a lower ability to identify their own accuracy in the perception of interoceptive signals, or, in other words, difficulty trusting bodily signals [21,22].

We are currently exposed to an excess of exteroceptive stimuli for food consumption, marked by the high availability of a wide variety of ultra-processed and hyperpalatable foods, in addition to increasingly larger food portions [23] that end up intensifying the reward responses and circumventing the homeostatic balance mechanisms [16]. With the accelerated rhythms of everyday life, little is observed of this relation between the sensory perception of food, its effects on bodily sensations and the way we eat, so that this effect of environmental stimuli on eating behaviour manifests itself mainly in an unconscious and thoughtless way [24].

The perception and appreciation of interoceptive sensations can be a way to reduce vulnerability to environmental stimuli, improve eating behaviour and prevent obesity [4]. It is known that individuals with greater interoceptive sensitivity tend to value the physiological signs of hunger and satiety as guides to food choices [25], and that they tend to be less impulsive and make better decisions [26], in addition to showing better emotion modulation [27,28] and being more efficient at integrating environmental sensory signals [29,30,31]. Strategies to increase interoceptive sensitivity have been evaluated, with promising (though preliminary) results after different types of interventions, such as mirror self-observation exercises [32], “power posing” practices [33], contingent biofeedback [34] and mindfulness meditation training [35].

In the field of food consumption, interventions focused on the attention and reconnaissance of exteroceptive stimuli have been recently studied, especially in interventions with children, although interventions with adult women have shown promising results for promoting better eating attitudes and behaviour [36,37]. Sensory experiences associated with information about the sensory aspects involved in eating experiences (sensory education) were used as a way to improve the relationship with food [38], with positive results in the ability to perceive and describe the sensory aspects of food [39,40], and also in eating behaviour patterns, including an increase in the number of food choices made in response to internal signals and not as a reflection of pre-established behaviour patterns [37,38], an increase in satisfaction when eating [36,37] and a decrease in food impulsivity [36].

This study evaluated women who experienced difficulty maintaining their body weight and who participated in the Food and Nutrition Education Program with Sensory and Cognitive Exercises (PESC), an intervention designed to promote consciousness of eating experiences [41]. The aim of this article is to evaluate the interoceptive sensitivity and the expression of exteroceptive perception before and after participating in the PESC. Our hypothesis is that exercises that promote the perception and the attribution of meaning to bodily sensations triggered by physiological states and environmental stimuli in different contemporary food contexts can result in an increase in interoceptive sensitivity and in exteroceptive stimuli processing. The joint assessment of interoception and exteroception in an intervention study highlights the integrated process that occurs between these two strands of sensoriality and is a pioneering characteristic of this work.

## 2. Materials and Methods

This study was approved by the Research Ethics Committee of the Clinic Hospital of Ribeirão Preto (HCRP-USP), code 3.335.083.

### 2.1. Participants and Study Design

The call for those interested in participating in the research was made through social media, e-mail and publication on the university’s website. We targeted adult women (aged between 20 and 59 years old), with a BMI between 18.5 and 34.9 kg/m^2^, who reported difficulty in maintaining body weight (evaluated by self-reported weight gain greater than 5% of the body weight in the previous 12 months) and who reported desire to improve their relationship with food. Women using psychotropic medications, smokers and women with BMI equal to or greater than 35 kg/m^2^ were excluded, as these conditions alter taste perception [42,43,44,45]. Pregnant women and breastfeeding women were excluded because of the food and behavioural specificities of these conditions. Nutritionists were excluded due to their proximity to the topic, which could represent a bias in this work. Finally, women with an allergy or intolerance to any of the foods used in the intervention were excluded.

Fifty-four women were screened and considered to be suitable for participation in the study. Participants were randomly allocated into two groups: intervention group (I-PESC) and control group (C-PESC). Twice as many participants were directed into I-PESC group due to the expectation of the greater occurrence of dropouts during the intervention. Participants were not aware of the group to which they belonged.

**Figure 1:**
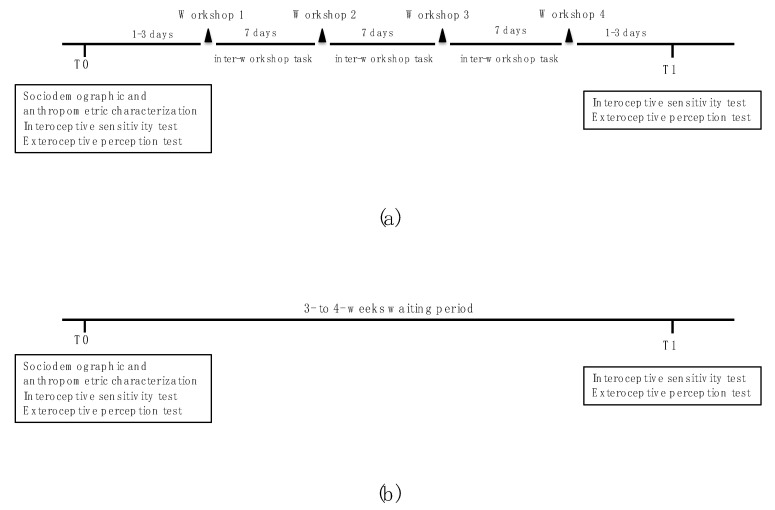
Study design in (**a**) intervention and (**b**) control conditions.

The participants in the I-PESC were evaluated at two moments: before the first PESC workshop (T0) and after the fourth PESC workshop (T1), with a 3- to 4-week interval between T0 and T1. The C-PESC group participants were evaluated at two moments (T0 and T1) with a 3- to 4-week interval (waiting period) between each evaluation (Figure 1).

Of the 36 participants allocated in I-PESC, 19 completed the assessment post-intervention. Dropouts occurred due to a lack of time to participate in the workshops. All the 18 participants allocated in C-PESC completed the second assessment.

### 2.2. PESC

The Food and Nutrition Education Program with Sensory and Cognitive Exercises (PESC) was developed with the intention to promote the consciousness of eating experiences. The detailed description of the activities and the application protocol were previously published [41].

The PESC consists of four 2 h workshops, and three inter-workshop exercises to be carried out at home, between each meeting. In the workshops, exercises inspired by everyday eating situations were applied in order to simulate triggering situations for food consumption. In these exercises, both exteroceptive and interoceptive aspects involved in the eating experience are explored, followed by the location of triggered body sensations, cognitive reflection on the topics covered and the connection with the food history and habits of each participant. Thus, sensory and cognitive aspects are explored in all workshops, followed by the attribution of sense/meaning to the proposed experience in order to promote an awareness of eating experiences [46,47].

The topics covered in each of the workshops are: (1) the senses and the desire to eat; (2) the senses and food pleasure; (3) hunger and satiety: how we deal with bodily signals; and (4) how we record experiences in the body.

### 2.3. Sociodemographic and Anthropometric Characterization

To characterize the participants, data regarding age, marital status, educational level, per capita income and self-reported weight variation (Kg) in the previous twelve months were collected. Participants’ body weight (Kg) was measured with a 50 g precision digital weight machine (Marte^®^) and height (m) with a 1 mm precision stadiometer (Cescorf^®^). All the measures were carried out according to a standard protocol [48].

### 2.4. Interoceptive Sensitivity

Interoceptive sensitivity was evaluated at T0 and T1 using the heart beat tracking task, as described by Schandry [49]. Participants were required to count their own heartbeats at intervals of 25, 35, 45 and 55 s, with an interval of 30 s between each count. The number of beats counted in each interval was compared with values obtained by a heart frequency monitor (Polar^®^ H10) whose validity and reliability, as compared to alternative ECG measurement devices, was already shown [50]. Later, the cardiac perception score was determined by the mean of the score in the four evaluated intervals:

$$Score=1-\frac{\left(recorded\;heartbeats-counted\;heartbeats\right)}{recorded\;heartbeats}$$

### 2.5. Exteroceptive Perception

To assess the perception of exteroceptive aspects during eating experience, a descriptive text on three foods was requested at T0 and T1. Participants received a snack consisting of coffee, salty biscuits and homemade lemon cake and had to write the text after tasting them. This assessment instrument is an adaptation drawn from other production text methods used in dietary interventions that explored the sensory aspects of eating experiences [38,40].

It is considered here that verbal expression is only possible after the conscious perception of the experiences [47] and that the number of terms used in the description of the food reflects the involvement and affectation of the participant in the activity [51].

### 2.6. Data Analysis

#### 2.6.1. Produced Text Analysis

The texts produced were transcribed and then content analysis was applied [52]. The software Atlas.ti, version 9.1.2 (ATLAS.ti Scientific Software Development GmbH, Berlin, Germany) was used for coding and categorizing the material.

First, the texts were segmented into meaning units, which are fragments of the text produced that carry a meaning—a piece of information transmitted by the participant—which may consist of a single word or a sequence of several words, depending on the structure of the text produced.

Then, the meaning units were coded and grouped into categories. The frequency of the meaning units in each category was then submitted to statistical analysis.

#### 2.6.2. Statistical Analysis

All variables underwent descriptive analysis.

For the analysis of sample characterization variables, Students’ *t*-test was used for independent samples and a 95% confidence interval was considered.

For comparison between the I-PESC and C-PESC groups at T0 or T1 and for the evaluation of the variation of I-PESC or C-PESC between T0 and T1, a linear regression model with mixed effects was used. This model considers a random effect per individual, considering that the individual has two measures taken (T0 and T1). The fixed effect represents the independence between the measures taken for each individual. The estimated differences with their respective *p* values and 95% confidence intervals are presented. Confidence intervals that do not include the zero value bring evidence of difference and their limits show the magnitude of this difference. Confidence intervals that encompass the zero value do not provide evidence of difference.

To verify the possible associations between the variation in interoception and the variation in the frequency of meaning units in the different categories and subcategories of the texts between T0 and T1, tables and contingency graphs were made with the division of participants between those who decreased and those who increased their interoceptive sensitivity after the intervention, followed by Fisher’s exact association test.

All statistical analyses were conducted using Statistical Analysis Software (version 9.3, SAS institute, Inc., Cary, NC, USA).

## 3. Results

### 3.1. Sample Characterization

The participants in this study were mostly single (*n* = 18, 49%) and had completed higher education (*n* = 29, 78%). The other data considered to characterize the groups are shown in Table 1. No differences were observed between the intervention and control groups in any aspect evaluated.

### 3.2. Interpceptive Sensitivity

The score achieved in the interoceptive sensitivity test on the evaluated moments by the intervention and control groups are shown in Table 2. The comparison of baseline values (T0) does not show any difference between the evaluated groups (*p* = 0.343). After participating in the PESC, the intervention group increased their interoceptive sensitivity, an effect not followed by the control group.

### 3.3. Exteroception Perception

Firstly, the meaning units were coded with the aim of generally characterizing the texts produced. The codification of the texts produced by both the intervention and control group illustrates how the participants expressed their perceptions about the food consumed (Table 3).

Next, the same meaning units were split into two categories: “Present experience” and “Repertoire”. The category “Present experience” refers to meaning units that describe some aspect of the food experience perceived during the tasting experience at the moment the test was performed (e.g., “The cake is tasty”), while the category “Repertoire” was for when the meaning unit brought an idea that already existed before the test was carried out, such as a baggage brought by the participant (e.g., “I like sweets”).

Finally, the category “Present experience” was subcategorized into “Object” or “Subject–object relationship”. The “Object” subcategory refers to the meaning units that deal with intrinsic aspects of the food tasted, such as the description of the food’s sensory attributes (e.g., “The cake is fluffy”). The subcategory “Subject–object relationship” refers to the meaning units that describe how the food, or some aspect of the food, affects the participant (e.g., “I like the sound it makes when chewing”).

Here, we consider that the category “Present experience” and its subcategories, “Object” and “Subject-to-object relationship”, are expressions of what was perceived during the tasting experience; that is, they are expressions of the exteroceptive perception.

**Figure 2:**
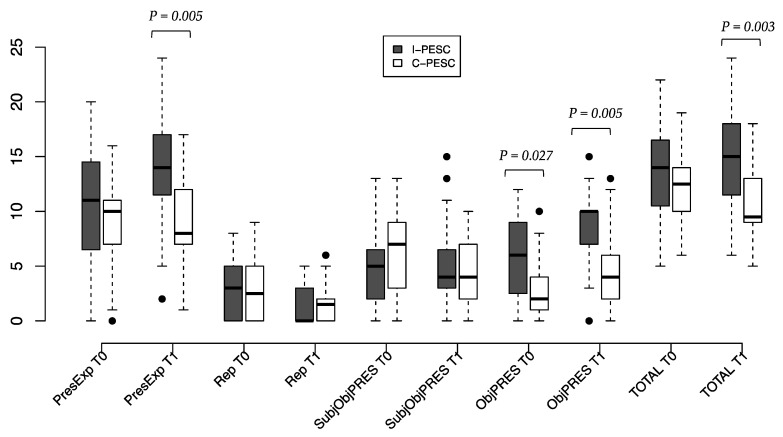
Frequency of meaning units in each category and subcategory at evaluated moments and groups. *p* < 0.05 indicates difference between groups. ● indicates outliers.

The frequency of meaning units in the categories “Present experience” (PresExp) and “Repertoire” (Rep), and in the subcategories “Subject–object relationship” (SubjObjPRES) and “Object” (ObjPRES), in the evaluated moments and groups are presented in Figure 2.

Baseline values (T0) indicate similarities between the I-PESC and C-PESC groups, with the exception of the “Object” subcategory, for which the I-PESC group had a higher frequency of meaning units at T0 (*p* = 0.027). After the intervention, the frequency of “Present experience” meaning units is higher in the I-PESC group, with an estimated average increase of 3.2 occurrences (*p* = 0.001), while no changes were observed in the C-PESC group. In the “Object” subcategory, the I-PESC group had an estimated average increase of 2.5 occurrences (*p* = 0.001), against an average increase of 1.4 occurrences (*p* = 0.016) in the C-PESC group. Finally, considering the total meaning units, the I-PESC group produced an average of 4.3 (*p* = 0.003) more meaning units than C-PESC group at T1.

### 3.4. Interoceptive Sensitivity X Exteroceptive Perception

Participants from the I-PESC group were split into two subgroups: participants whose interoceptive sensitivity increased (T1-T0 > 0) after participating in PESC (*n* = 11) and participants whose interoceptive sensitivity decreased (T1-T0 < 0) after participating in PESC (*n* = 6). Contingency tables were then constructed, considering the variation of the meaning units’ frequency in the categories “Present experience” and “Repertoire” and in the subcategories “Object” and “Subject–object relationship” in each subgroup.

**Figure 3:**
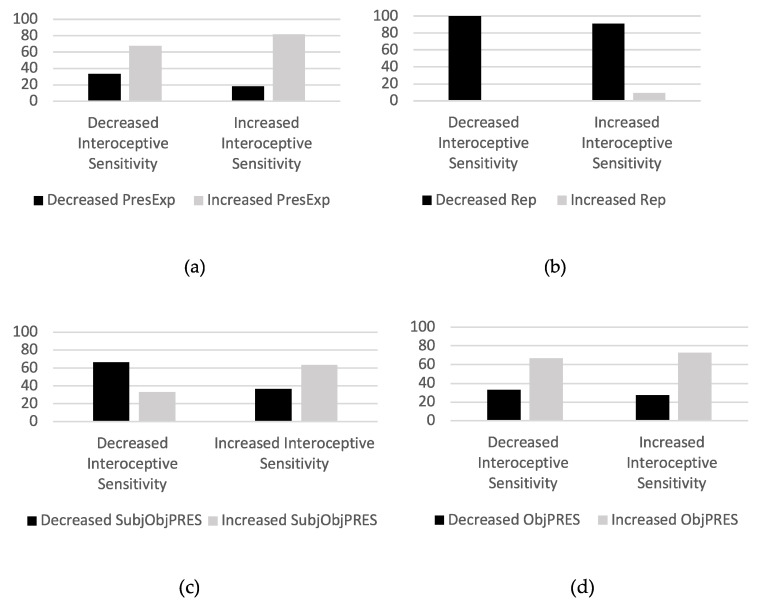
Contingency graphics. Participants in the I-PESC group were divided into two subgroups: those who increased their interoceptive sensitivity (T1-T0 > 0) and those who decreased their interoceptive sensitivity (T1-T0 < 0). In each subgroup, it is shown the percentage of participants who: (**a**) increased or decreased the frequency of meaning units in the “Present experience” category; (**b**) increased or decreased the frequency of meaning units in the “Repertoire” category; (**c**) increased or decreased the frequency of meaning units in the “Subject–object relationship” subcategory; and (**d**) increased or decreased the frequency of meaning units in the “Object” subcategory.

Fisher’s exact test showed no association between interoceptive sensitivity variation and variation of the meaning units’ frequency in any of the categories or subcategories. Despite that, the contingency graph (Figure 3) shows that most participants who increased their interoceptive sensitivity after the intervention also increased the frequency of meaning units in the subcategory “Subject–object relationship”, while participants who decreased their interoceptive sensitivity also mostly decreased the frequency of meaning units in the category “Subject–object relationship”, indicating a possible association between these two variables.

## 4. Discussion

The increase in interoceptive sensitivity observed with the heartbeat tracking task is a very positive result of this study. In the literature, the variation of this parameter is evaluated after very diversified interventions, both due to the characteristics of the intervention, which include breathing exercises [53], body scanning practices [34,53,54], biofeedback techniques [34], power posing techniques [33], self-focused attention [55], among others, as well as the duration of these interventions, which range from occasional practices of a few minutes duration [34,55] to more intense interventions, with daily practices [33], or over several weeks [54], which is probably related to the great variability of the results found.

The PESC activities [41] do not strictly fit into any of the interventions mentioned above, but they include exercises that promote attention and reflection to the sensory aspects involved in the eating experience and their effects on bodily sensations, including the perception and location of bodily sensations triggered by the interaction with food in different contexts. Such activities, divided into four 2 h meetings over 3 to 4 weeks, with weekly exercises between each meeting, are indicated to be effective at increasing interoceptive sensitivity.

As for the texts produced, the observed increment of meaning units used in the description of food after the intervention is consistent with other intervention studies that also applied attention exercises and explorations of the sensory aspects of food [38,40]. The greater frequency of total meaning units showed by the I-PESC group when compared to the C-PESC group at T1, and in particular the increase in the frequency of meaning units in the “Present experience” category showed by the I-PESC group, may indicate a greater involvement in the activity and a greater awareness of how consumed foods stimulate and affect the individual [51].

As far as it can be verified, this is the first study that evaluated, in the same intervention, the variations in interoceptive sensitivity and in the expression of exteroceptive perception. The suggested association between increased interoceptive sensitivity (the accuracy of physiological bodily signal perception) and the increased frequency of meaning units in the “Subject–object relationship” subcategory (textual production relating to how food affects the participant in the moment of the tasting experience), indicates that there is a possible connection between the increment mechanisms of these two parameters. It is known that interoception is related to how we represent and perceive our body as the subject of experiences [56], in addition to being fundamental in the integration of external multisensory stimuli [29]. The results of this work indicate a possible extrapolation of these findings, as they point to a possible associated modulation of these parameters through an intervention with sensory and cognitive exercises.

The small sample size is an important limitation of this work, meaning that subsequent studies involving a larger number of participants can explore in greater depth the effects of applying sensory and cognitive exercises on the perception and expression of bodily sensations in their interoceptive and exteroceptive strands. The inclusion of a third assessment moment, a few weeks after the completion of the intervention, could also provide information on how these effects behave over time.

An important point to be explored in future works is the extent to which the increase in interoceptive sensitivity and the increase in the expression of exteroceptive stimuli’s perception can contribute to the strengthening of self-regulatory mechanisms, which can make individuals less vulnerable to exaggerated food consumption triggered by environmental stimuli. There are many cross-sectional studies in the literature that relate difficulty in identifying, valuing and trusting bodily signals to eating disorders [20,57,58,59], greater eating impulsiveness [60], emotional eating [61] and obesity [62], which makes interventions capable of improving interoception appear very promising with regard to the management of these conditions [4,63].

## 5. Conclusions

The results of this study showed that the PESC promoted an increase in interoceptive sensitivity and in the expression of exteroceptive perception in women who had difficulty controlling their body weight. These results bring important contributions in the search for strategies to promote the perception of bodily sensations in the face of physiological and environmental food stimuli. More studies are needed to assess the long-term effects of interventions with sensory and cognitive exercises on interoception and exteroception, as well as the possible effects on improving eating behaviour and body weight management.

## Figures and Tables

**Table 1:** Sample characterization.

	I−PESC (*n* = 19)	C−PESC (*n* = 18)	*p*	95% Confidence Interval (Group Difference)
Age (years)	36.78 ± 12.73	36.00 ± 12.53	0.850	−7.6; 9.2
Income (BRL)	3752.78 ± 3012.54	4117.65 ± 2232.78	0.685	−2185.4; 1455.7
Body weight (Kg)	73.31 ± 9.08	75.26 ± 13.98	0.620	−9.9; 6.0
BMI (Kg/m^2^)	28.18 ± 3.19	26.44 ± 4.42	0.181	−0.8; 4.3
Body weight variation (Kg)	5.36 ± 1.86	7.03 ± 3.65	0.096	−3.6; 0.3
Body weight variation (%)	8.05 ± 2.93	10.12 ± 4.83	0.128	−4.7; 0.6

I-PESC = intervention group; C-PESC = control group. BRL = Brazilian real. Results expressed as mean ± standard deviation.

**Table 2:** Interoceptive sensitivity test scores at T0 and T1.

	T0	T1	Variation	95% Confidence Interval (Variation)
I-PESC	0.64 ± 0.17	0.73 ± 0.14	0.08	0.02; 0.14
C-PESC	0.69 ± 0.13	0.68 ± 0.13	−0.02	−0.03; 0.08

I-PESC = intervention group; C-PESC = control group. Results expressed as mean ± standard deviation. A linear regression model with mixed effects was applied. Confidence intervals that do not include the zero value bring evidence of difference and their limits show the magnitude of this difference. Confidence intervals that encompass the zero value do not provide evidence of difference.

**Table 3:** Coding of the produced texts.

Category Group	Category	Code	Example
Senses	Sense perception	Aroma	“Biscuit smell”
		Taste/Flavour	“Not too salty”
		Vision	“Golden”
		Texture	“Very fluffy texture”
		Temperature	“Hot coffee”
		Sound	“Makes crunchy sound”
	Sense evaluation	Aroma	“I love the smell of coffee”
		Taste/Flavour	“Only the touch of butter doesn’t please me”
		Vision	“Pretty”
		Texture	“It moistens as I chew it in a very pleasant way”
		Temperature	“The warm feeling makes it very pleasant”
		Sound	“It does the *crec-crec* that I love”
	Overall food evaluation	Hedonic relationship	“Tasty”
		Context	“Matches with coffee and cake”
	What food triggers in the subject	Memory	“Remind me of the time I went on a diet”
		Bodily sensations	“The feeling of drinking it is very good”
		Emotions	“Makes me happier”
		Desire	“...but it wouldn’t satisfy the desire to eat a sweet”
	Other aspects	Attitude	“If the coffee was good, maybe I would finish the biscuit by drinking more coffee”
		Convenience/Utility	“It’s a very practical food”

## Data Availability

The data presented in this study are openly available in Mendelay Data at http://dx.doi.org/10.17632/8b29ftftz7 (accessed on 31 November 2021).
